# 1385. Impact of Deceased Organ Donor Injection Drug Use on Donor Culture Positivity

**DOI:** 10.1093/ofid/ofab466.1577

**Published:** 2021-12-04

**Authors:** Krista Moore, Ebbing Lautenbach, Emily Blumberg, Jennifer Han, Dong Heun Lee, Heather Clauss, Richard Hasz, Warren Bilker, Antonette Climaco, Esther Molnar, Darcy Alimenti, Sharon West, Pam C Tolomeo, Judith A Anesi, Judith A Anesi

**Affiliations:** 1 Perelman School of Medicine, University of Pennsylvania, Philadelphia, Pennsylvania; 2 University of Pennsylvania, Philadelphia, Pennsylvania; 3 GlaxoSmithKline, Rockville, MD, Rockville, Maryland; 4 University of California San Francisco, San Francisco, California; 5 Lewis Katz School of Medicine, Temple University, Philadelphia, PA; 6 Gift of Life Donor Program, Philadelphia, PA; 7 Albert Einstein Medical Center, Philadelphia, PA; 8 Bass Medical Group, Philadelphia, PA; 9 University of Pennsylvania Perelman School of Medicine, Philadelphia, PA

## Abstract

**Background:**

With the ongoing opioid epidemic in the US, there has been an increase in the proportion of deceased organ donors with a history of injection drug use (IDU), raising concern for additional infectious risks to transplantation.We sought to determine how recent IDU among deceased organ donors impacted donor culture results.

**Methods:**

A retrospective cohort study was conducted at four transplant centers in Philadelphia between 1/1/2015 and 6/30/2016. All deceased organ donors who donated ≥ 1 organ to one of the centers were included. Exposed donors were those with a recent history of IDU (defined by use in the prior 12 months based on donor chart review). Unexposed donors were those with no recent history of IDU. The primary outcome was any positive donor culture (taken during the terminal hospitalization or at the time of organ procurement) for bacteria or *Candida.* Multivariable logistic regression was used to determine the association between recent IDU and donor culture positivity. Secondarily, the association between donor IDU and isolation of (1) a multidrug-resistant organism (MDRO) on culture, (2) *Staphylococcus aureus* on culture, (3) *Candida* on non-respiratory culture, and (4) bacteria or *Candida* on blood culture were determined.

**Results:**

Of 394 total donors, 66 (17%) had a history of recent IDU and 343 (87%) had at least one positive donor culture. On multivariable analysis, recent IDU was associated with significantly increased odds of having at least one positive donor culture (aOR 3.6, 95% CI 1.1-11.9, *P*=0.04) and *Candida* on non-respiratory culture (aOR 2.6, 95% CI 1.1-6.2, *P*=0.03). However, recent IDU was not significantly associated with increased odds of MDRO on culture (OR 0.90, 95% CI 0.41-1.93, *P*=0.79), *S. aureus* on culture (OR 1.35, 95% CI 0.79-2.28, *P*=0.27), or positive blood culture (OR 0.79, 95% CI 0.32-1.95, *P*=0.60).

**Conclusion:**

Donors with a recent history of IDU are more likely to have bacteria or *Candida* identified on cultures taken during their terminal hospitalization or at organ procurement. This increase does not appear to be driven by MDROs, *S*. *aureus*, or bloodstream infections but rather by *Candida* isolated from non-respiratory sites, potentially alleviating some fears surrounding the acceptance of solid organs from donors with a history of recent IDU.

**Disclosures:**

**Ebbing Lautenbach, MD, MPH, MSCE**, **Merck** (Other Financial or Material Support, Member of Data and Safety Monitoring Board (DSMB)) **Emily Blumberg, MD**, **Amplyx** (Other Financial or Material Support, Member of Data and Safety Monitoring Board (DSMB))**Hologic** (Research Grant or Support)**Merck** (Grant/Research Support, Other Financial or Material Support, Member of Scientific Advisory Committee)**Takeda** (Research Grant or Support, Other Financial or Material Support, Member of Scientific Advisory Committee) **Jennifer Han, MD, MSCE**, **GlaxoSmithKline** (Employee, Shareholder) **Judith A. Anesi, MD, MSCE**, Nothing to disclose

